# Low Resolution Structural Studies Indicate that the Activator of Hsp90 ATPase 1 (Aha1) of *Leishmania braziliensis* Has an Elongated Shape Which Allows Its Interaction with Both N- and M-Domains of Hsp90

**DOI:** 10.1371/journal.pone.0066822

**Published:** 2013-06-24

**Authors:** Thiago V. Seraphim, Marina M. Alves, Indjara M. Silva, Francisco E. R. Gomes, Kelly P. Silva, Silvane M. F. Murta, Leandro R. S. Barbosa, Júlio C. Borges

**Affiliations:** 1 Instituto de Química de São Carlos, Universidade de São Paulo - USP, São Carlos, SP, Brazil; 2 Centro de Ciências Biológicas e da Saúde, Universidade Federal de São Carlos, São Carlos, SP, Brazil; 3 Centro de Pesquisa René Rachou, Fiocruz, Belo Horizonte, MG, Brazil; 4 Departamento de Física Geral, Instituto de Física, Universidade de São Paulo - USP, São Paulo, SP, Brazil; Institut Pasteur, France

## Abstract

The Hsp90 molecular chaperone is essential for protein homeostasis and in the maturation of proteins involved with cell-cycle control. The low ATPase activity of Hsp90 is critical to drive its functional cycle, which is dependent on the Hsp90 cochaperones. The Activator of Hsp90 ATPase-1 (Aha1) is a protein formed by two domains, N- and C-terminal, that stimulates the Hsp90 ATPase activity by several folds. Although the relevance of Aha1 for Hsp90 functions has been proved, as well as its involvement in the desensitization to inhibitors of the Hsp90, the knowledge on its overall structure and behavior in solution is limited. In this work we present the functional and structural characterization of *Leishmania braziliensis* Aha1 (LbAha1). This protozoan is the causative agent of cutaneous and mucocutaneous leishmaniasis, a neglected disease. The recombinant LbAha1 behaves as an elongated monomer and is organized into two folded domains interconnected by a flexible linker. Functional experiments showed that LbAha1 interacts with *L. braziliensis* Hsp90 (LbHsp90) with micromolar dissociation constant in a stoichiometry of 2 LbAha1 to 1 LbHsp90 dimer and stimulates 10-fold the LbHsp90 ATPase activity showing positive cooperativity. Furthermore, the LbHsp90::LbAha1 complex is directed by enthalphy and opposed by entropy, probably due to the spatial freedom restrictions imposed by the proteins’ interactions. Small-angle X-ray scattering data allowed the reconstruction of low resolution models and rigid body simulations of LbAha1, indicating its mode of action on LbHsp90. Western blot experiments allowed Aha1 identification (as well as Hsp90) in three Leishmania species at two temperatures, suggesting that Aha1 is a cognate protein. All these data shed light on the LbAha1 mechanism of action, showing that it has structural dimensions and flexibility that allow interacting with both N-terminal and middle domains of the LbHsp90.

## Introduction

The molecular chaperones of the Hsp90 family are essential for the growth of many organisms [1], [2], including protozoans such as *Plasmodium falciparum, Leishmania donovani* and *L. amazonensis* where they work in the heat stress response for the cellular differentiation in the parasite life cycle [3]–[7]. In addition, this protein family assists in the protein folding and mediates protein homeostasis. The Hsp90s are among the most abundant proteins in unstressed cells (∼2%) (reviewed in [2], [8], [9]).

The Hsp90 are 82–96 kDa proteins that form homodimers where each protomer can be divided into 3 domains as follow: N-terminal domain (ND), middle domain (MD) and C-terminal dimerization domain [1], [2]. The Hsp90 ND has an ATP binding site and presents a weak ATPase activity [1], [2]. This domain can also bind to client proteins, Hsp90 cochaperones and some Hsp90 inhibitors, such as geldanamycin, 17-AAG and radicicol. Besides, the Hsp90 ND can also dimerize during the Hsp90 functional cycle [1], [2], [10]. The Hsp90 MD participates of the ATPase activity of the Hsp90 ND and interacts with client proteins and Hsp90 cochaperones [11]. It should be highlighted that the modulation of the Hsp90 ATPase activity by its cochaperone interactions can compromise the inhibitor therapeutic response of Hsp90 [12]. It is suggested that the binding of some Hsp90 cochaperones, such as Aha1 (Activator of Hsp90 ATPase 1), can reverse, *in vivo,* the Hsp90 inhibitor effects [13].

The Hsp90 ATPase cycle is assisted by various cochaperones [2], one of which is the Aha1, which has molecular mass of around 38 kDa and binds to the Hsp90 MD [11]. The intrinsic ATPase activity of Hsp90 in relatively weak [14]–[16] and Aha1 cochaperone can stimulate it, indicating an important role in the Hsp90 ATP-driven cycle [2], [17]. The ATPase function of the Hsp90 depends on the Arg380 (numbering of yeast Hsp90) in the loop (370–390) located in the Hsp90 MD [11], [18] and the Aha1 interaction assists in the Arg380 stabilization in the ATP γ-phosphate coordination process [19].

Aha1 can be divided into two domains, N- and C-terminal [11], [20], [21]. However, in spite of the availability of tridimensional structures of the yeast Aha1 (yAha1) N-terminal domain [19] and of the human Aha1 (hAha1) C-terminal domain (PDB: 1X53), understanding the functionality of each domain is limited. Although the full length yAha1 is needed to promote a maximum stimulation of Hsp90 ATPase activity, it is known that a considerable stimulation can be obtained with high concentrations of the yAha1 N-terminal domain, suggesting that this region is responsible for the Hsp90 ATPase activity stimulation [17], [19], [21]. However, it has been shown that the isolated C-terminal domain is also able to stimulate the Hsp90 ATPase activity, while the N-terminal domain only binds to the Hsp90 MD [20]. The core of Aha1-Hsp90 interaction lies on the Aha1 N-terminal domain and Hsp90 MD, but the Aha1 C-terminal domain can interact with the Hsp90 ND in its dimerized state [19], [22]. While some works has shown that the N-terminal domain of hAha1 binds to the Hsp90 MD to stimulate its ATPase activity, other studies have indicated the Aha1 C-terminal domain is also required for that stimulation, and both domains bind cooperatively to the Hsp90 dimer interface [20], [22].

Considering that little is known about the Aha1 structure, mainly in protozoan, and the relevance of this protein to the Hsp90 functional cycle, we present the biochemical and biophysical characterization of *Leishmania braziliensis* Aha1 (LbAha1). This protozoan is the causative agent of cutaneous and mucocutaneous Leishmaniasis, which according to the World Health Organization, it is a neglected disease [23], [24]. The drugs currently used in chemotherapy have various deficiencies, such as toxicity, high cost and emerging resistance [25]. Consequently, the development of novel drug targets is an urgent priority. Here, we present data showing that LbAha1 is composed of two domains that are organized in a high elongated protein. Through small angle X-ray scattering (SAXS) data, homology molecular modeling, *ab initio* modeling and rigid body simulation, we propose a structural organization for LbAha1. This model suggests that LbAha1 can interact with both MD and ND of *L. braziliensis* Hsp90 (LbHsp90). LbAha1 was able to stimulate the weak ATPase activity of LbHsp90 by around 10-fold exhibiting a cooperative behavior according to the model that two LbAha1 molecules can act on one LbHsp90 dimer. Additionally, Aha1 and Hsp90 were identified in three Leishmania species (including *L. braziliensis*) at two growth temperatures, suggesting that Aha1 as well as Hsp90 are cognate proteins.

## Materials and Methods

### Sequence Analysis and Homology Molecular Modeling

LbAha1 amino acid sequence (GenPept ID: XP_001563948.1) was aligned with yAha1 (GenPept ID: Q12449.1) and hAha1 (GenPept ID: O95433.1) using the Clustal W program (http://www.ebi.ac.uk/Tools/msa/clustalw2/). The Sednterp program (http://www.jphilo.mailway.com/download.htm) was used to estimate some of the LbAha1 physicochemical parameters. The homology-modeling of LbAha1 N- and C-terminal domains were automatically built by the Swiss Model server [26] using as templates the structures of the yAha1 N-terminal domain (PDB: 1USV) and hAha1 C-terminal domain (PDB: 1X53). The overall stereochemical quality of the LbAha1 N- and C-terminal domains were investigated by the Procheck [27] and Verify 3-D programs [28].

### Cloning, Expression and Purification

The DNA sequence coding for LbAha1 was amplified by PCR from *L. braziliensis* genomic DNA using the specific primers: 5′-AATCATATGGCTAAGGTCGGCGAGG-3′ and 5′-ATAGAATTCAGATGTACTCGAGGGAG-3′. The PCR product was inserted in the pET23a vector between the *Nde* I and *EcoR* I restriction sites, yielding the pET23a::LbAha1 expression vector. The cloning process was verified by automatic DNA sequencing.

The recombinant LbAha1 was produced in *Escherichia coli* cells Bl21(DE3) pLysS strain at 30°C (200 rpm) by 0.1 mM of IPTG for 4 h. After centrifugation, the cells were resuspended in the buffer 25 mM Tris-HCl (pH 8.0), 20 mM NaCl, 2 mM EDTA and 20 µg.mL^−1^ of lysozyme (Sigma) and 5 units of DNase (Promega). After 30 min of incubation on ice, the cells were disrupted by sonication and centrifuged for 20 min at 20,000 x g. The supernatant was filtered using a 0.45 µm filter membrane and submitted to an ion exchange chromatography, using a HighQ Support column (Bio-Rad) in the buffer 25 mM Tris-HCl (pH 8.0), 20 mM NaCl, 2 mM EDTA. LbAha1 was eluted with a linear gradient of buffer 25 mM Tris-HCl, 500 mM NaCl, 2 mM EDTA. The fractions containing LbAha1were dialyzed overnight against 10 mM sodium phosphate (pH 7.4) buffer and submitted to a calcium affinity chromatography in a Ceramic Hydroxyapatite Type II (Bio-Rad) resin. LbAha1 elution was performed by a linear gradient of 10–500 mM sodium phosphate (pH 7.4). The final step of LbAha1 purification was done by preparative size exclusion chromatography (SEC), using a Superdex 200 16/60 pg column (GE Healthcare), previously equilibrated with the 25 mM sodium phosphate (pH 7.0) buffer, containing 50 mM NaCl, 2 mM EDTA and 1 mM β-mercaptoethanol. All purification steps were performed using an Äkta Prime device (GE Healthcare). LbHsp90 purification was performed as previously described [16]. The protein concentrations were measured under denaturing conditions.

### Spectroscopy Studies

Circular dichroism experiments were carried out in a J-810 spectropolarimeter (Jasco) coupled to a Peltier-type system for temperature control. The CD spectra were collected in a 0.2 mm path length quartz cell containing 0.5 mg.mL^−1^ of LbAha1 in the 25 mM sodium phosphate (pH 7.0) buffer, which contained 50 mM NaCl, 2 mM EDTA and 1 mM β-mercaptoethanol. The secondary structure estimation for LbAha1 was performed using the DichroWeb server [29]. LbAha1 chemical-induced unfolding, followed by CD signal at 220 nm, was carried out using a 1 mm path length quartz cuvette with 0.25 mg.mL^−1^ of protein in the same buffer, as described earlier, after incubation at room temperature for 1 hour. All CD data were normalized to residual molar ellipticity ([θ]).

The intrinsic fluorescence emission measurements were performed in an F-4500 fluorescence spectrophotometer (Hitachi) at 20°C. The fluorescence emission spectra of 5 µM of LbAha1 in the buffer described above were read in a 1×0.2 cm quartz cuvette after sample excitation at 280 nm. The chemical-induced unfolding experiments followed by intrinsic fluorescence emission were performed with LbAha1 5 µM prepared in the buffer described above containing the indicated denaturant agent. The fluorescence emission spectra were recorded after incubation at room temperature for 1 hour and the data were quantified by the center of spectral mass (<λ>), as follow:
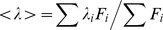
(1)where, *Fi* is the fluorescence intensity measured at each wavelength (*λi*).

The chemical-induced unfolding data of both CD and fluorescence data were fitted by a sigmoidal double-Boltzmann function in order to obtain the C_m_-values for each transition, which is the chemical-concentration of the midpoint transition.

### Hydrodynamic Experiments

Analytical SEC (aSEC) experiments were conducted as previously described [16] using the column Superdex 200 10/300 GL (GE Healthcare) equilibrated in the 25 mM sodium phosphate (pH 7.0) buffer, containing 50 mM NaCl, 2 mM EDTA and 1 mM β-mercaptoethanol.

Sedimentation velocity experiments were performed in an Optima XL-A analytical ultracentrifuge (Beckman) with the AN-60 Ti rotor set at 30,000 rpm at 20 ^o^C. The LbAha1 sedimentation data were monitored by absorbance at 286 nm and the experiments were performed with protein concentrations of 300 to 1,000 µg.mL^−1^ solved in the buffer described above. The AUC data were treated by the SedFit 12.2 software [30] using the frictional ratio (*ƒ/ƒ*_0_) as a regularization parameter, which was allowed to float freely. The *s*-values, obtained from the peak of the continuous c(S) distributions, were normalized to standard conditions (*s_20,w_* – sedimentation coefficient at 20°C, in water). Buffer density (1.00369 g.mL^−1^) and viscosity and (0.00102 Poise), and the partial specific volume of the LbAha1 (0.7346 mL.g^−1^) were estimated by the Sednterp program (http://www.jphilo.mailway.com/download.htm). The *s^0^_20,w_*-value (*s_20,w_* at 0 mg.mL^−1^ of protein) was estimated using the *s_20,w_* graph as a function of protein concentration, which is an intrinsic property of the particle [31].

### Isothermal Titration Calorimetry Experiments

Isothermal titration calorimetry (ITC) experiments were performed at range temperature of 20^o^C to 37^o^C in an iTC200 microcalorimeter (GE Healthcare Life Sciences). Both LbHsp90 and LbAha1 were dialyzed extensively against the 40 mM HEPES buffer (pH 7.5) containing 5 mM KCl. Twenty-three aliquots of 1.65 µL of LbAha1 at 130–150 µM were injected into 203.8 uL of LbHsp90 at around 5.5 µM (considering dimeric species). The enthalpy change for each injection was calculated by integrating the area under the peaks of the recorded time course of the change of power. The data were analyzed by the Microcal Origin software using the One Set of Sites curve fitting model in order to calculate the apparent binding enthalpy change (ΔH_app_), binding stoichiometry (n), and association constant (K_A_). The heat of injectant dilution was determined from the baseline at the end of titration and subtracted of the data. The apparent Gibbs energy (ΔG_app_) and apparent binding entropy change (ΔS_app_) were calculated by the following equation:
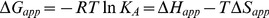
(2)Where T is the absolute temperature (in Kelvin) and R is the gas constant (in cal.K^−1^.mol^−1^). The apparent binding heat capacity change (ΔCp_app_) was estimated by the dependence on the ΔH_app_ with the temperature (Equation 3).
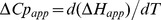
(3)


### ATPase Measurements

The ATPase activity measurements were performed spectrophotometrically by using the EnzChek Phosphate Assay kit (Invitrogen) as previously shown [16]. Summarily, LbHsp90 (1 µM of dimers) was incubated, at 37 ^o^C, with 0.5 mM of ATP in the presence of increasing concentrations of LbAha1 (0–20 µM). All samples were prepared in 40 mM HEPES (pH 7.5) buffer, containing 5 mM KCl. The hydrolyzed P_i_ was quantified as described above and the ATP hydrolysis rate was converted into relative ATPase activity.

### Small Angle X-ray Scattering

The LbAha1 SAXS data collection was performed at the D02A-SAXS2 beamline in the Laboratório Nacional de Luz Síncrotron (LNLS, Campinas-SP, Brazil). The X-ray scattering data were recorded using a two-dimensional position-sensitive MARCCD detector. The measurements were done with a monochromatic X-ray beam (λ = 1.488 Å) and a sample-to-detector distance of ∼1000 mm, which corresponds to the scattering vector range of 0.015< q <0.35 Å^−1^, where q is the magnitude of the q-vector defined by q = (4π/λ).sin θ (2θ is the scattering angle). The samples were measured in a 1 mm path length cell formed by two mica windows and the scattering patterns were collected in 300 s frames at several protein concentrations (1.1 mg.mL^−1^, 1.9 mg.mL^−1^ and 3.2 mg.mL^−1^ in the buffer 25 mM Sodium phosphate (pH 7.0), 50 mM NaCl, 2 mM EDTA, 1 mM β-mercaptoethanol). The SAXS curves were corrected for detector response and scaled by the incident beam intensity and the attenuation of the sample. The corrected buffer SAXS curve was subtracted from the sample scattering. The SAXS curves were normalized by the protein concentration and data quality, as well as Rg and Porod’s volume, were calculated by the Primus program [32]. GNOM software [33] was used for calculating the particle distance distribution function, p(r).

It is well-known that the scattering intensity can be represented by a Gaussian-like function in the small q-range, such approximation is known as Guinier’s law [34]. According to this methodology it is possible to evaluate the scattering particle radius of gyration, R_g_, and the forward scattering intensity, I(q→0), which is related to the protein molecular weight [35], [36].

The LbAha1 shape *ab initio* models (DAM model) were constructed using dummy atoms in a simulated annealing method performed by the DAMMIN program [37]. To compute the most probable *ab initio* model, a minimum of 10 individual runs were used and averaged using DAMAVER software [38]. Due to the LbAha1 domain organization, a multi-domain simulation was performed using the Ensemble Optimization Method (EOM) approach [39], applying the LbAha1 individual domain structures, which were obtained by molecular homology. This ensemble optimization involves 2 steps: 1) generation of 10,000 random conformers of the interconnected domains using the RanCh program; 2) the GAJOE program was used for selecting the best conformers that have an ensemble scattering curve consistent with experimental SAXS data, based on the smallest χ-value.

The DAM model and all EOM selected conformers, which presented the best ensemble among 10,000 conformers, were analyzed by the HydroPro software [40] in order to predict their hydrodynamic properties. The parameters of MM (38 kDa) and V_bar_ (0.7346 cm^3^.g^−1^) were estimated from the amino acid sequence of LbAha1 using the Sednterp software, as well as the parameters ρ and η (for standard conditions), at the temperature of 20 ^o^C.

### Parasites Growth, Protein Extraction and Western Blot Analysis

In this study, we used promastigote forms of three different New World *Leishmania* species: *L. (V.) braziliensis* (MHOM/BR/75/M2904); *L. (V.) guyanensis* (IUMB/BR/85/M9945) and *L. (L.) infantum chagasi* (MHOM/BR/74/PP75). Parasites were grown at 26 ^o^C in M199 medium supplemented with 10% heat-inactivated fetal calf serum, 50 µg.mL^−1^ gentamicin, 40 mM HEPES (pH 7.4), 1 µg.mL^−1^ biotin, 14 µg.mL^−1^ hypoxanthine, 0.36 mg.mL^−1^ sodium bicarbonate, 0.1 mM adenine, 6 µM biopterin and 250 µg.mL^−1^ hemin.

Parasites in the logarithmic growth phase (around 10^7^ parasites.mL^−1^ of culture) were divided into two tubes containing 30 mL of culture and incubated at 26°C or 37°C. After 2 h, 4 h and 6 h of incubation, the parasite aliquots (10 mL) were collected and centrifuged at 800×g, 4°C for 10 min and washed three times with RPMI medium (Sigma). The cell pellets were suspended in the 20 mM Tris-HCl buffer (pH 8.0) containing 5 mM NaCl, 1% Nonidet P-40 and protease inhibitor cocktail tablets (Roche), followed by incubation on ice for 10 min. Three freeze-thaw cycles were done to disrupt the parasites (N_2_: −196°C – Water bath: 37°C), the samples were centrifuged at 350×g, 4°C for 10 min, and the supernatant was stored at −70°C until use.

Western blot analysis were carried out in a nitrocellulose 0.22 µm membrane using rabbit-produced polyclonal antibodies against recombinant LbHsp90 (1∶2,000) and LbAha1 (1∶250) proteins (Célula B – Serviço de Produção de Anticorpos), and the monoclonal anti-α-tubulin mouse-produced antibody (1∶30,000) (clone B5-1-2, Sigma). The alkaline phosphatase-conjugated secondary antibodies were goat anti-mouse IgG (1∶30,000) and goat anti-rabbit IgG (1∶30,000) (Sigma).

## Results and Discussion

### LbAha1 Shares Low Sequence Identity with Orthologous Proteins

Aha1 was first identified in yeast and was identified in *S. pombe*, *A. thaliana*, *D. melanogaster*, *C. elegans* and *H. sapiens* [17]. LbAha1 has 23% and 30% of identity with the yAha1 and hAha1, respectively (Fig. S1– Supporting Information) suggesting low conservation degree. The Aha1 and Hsp90 proteins interact mainly by contacts involving the Aha1 N-terminal domain and the Hsp90 MD [19], [21], but there are additional contacts in the Aha1 C-terminal and the Hsp90 [20], [22]. The low conservation between LbAha1 and orthologous proteins suggests that the mechanism of interactions of LbAha1 with LbHsp90 can present some peculiarities.

**Figure 1:**
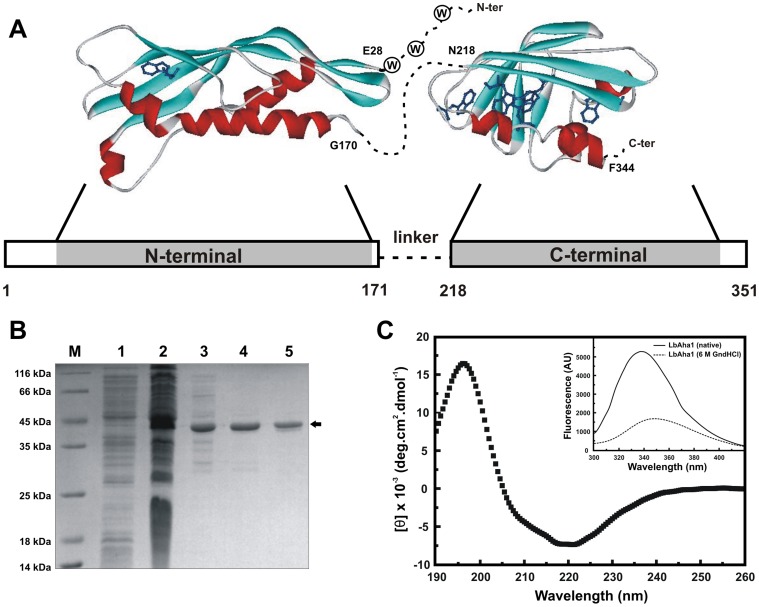
Structural features, acquisition and spectroscopic analysis of the LbAha1. **A)** Through homology-modeling, the LbAha1 domains were modeled using as templates the N- and C-terminal domains of the yAha1 (PDB: 1USV) and hAha1 (PDB: 1X53), respectively. The bar scheme represents the domain organization of LbAha1 and the regions highlighted in gray are represented by the structural domains shown. The first residues of the LbAha1 N-terminal domain, as well as the linker region and the last residues of the C-terminal domain, are represented by dotted lines. LbAha1 has 9 Trp residues along its structure (4 in the N-terminal domain and 5 in the C-terminal domain), which are shown as blue sticks, and at the beginning of the N-terminal domain, an absent and unstructured region in the template, the Trp residues are represented by circles. **B)** The LbAha1 protein was expressed in *E. coli* cells and purified by three chromatographic steps. All procedures were followed by SDS-PAGE, and the final purity of the target protein was higher than 95%. M: MM marker; 1 and 2: cell lysate before and after induction; 3, 4 and 5: LbAha1 after the anionic exchange, calcium affinity chromatography and preparative SEC, respectively. **C)** The CD spectra of LbAha1 were collected in 25 mM sodium phosphate (pH 7.0), 50 mM NaCl, 2 mM EDTA, 1 mM β-mercaptoethanol. The LbAha1 CD spectrum was compatible with those for proteins constituted by α-helix and β-sheet conformation, as estimated by the DichroWeb server (see text for details). *Inset*: Intrinsic fluorescence spectra of the LbAha1 at native (solid line) and denatured (dashed line) condition were acquired in the same buffer of the CD analysis, but the latter contained 6 M of GndHCl. Moreover, the recombinant LbAha1 protein was purified in the folded state.

In spite of the low sequential identity, the available structures of the yAha1 N-terminal domain (22% of identity) and hAha1 C-terminal domain (32% of identity) were used as templates for LbAha1 comparative-modeling (Fig. 1A). The generated structures were validated using the Procheck and Verify 3-D programs and both N- and C-terminal domains presented sufficient stereochemical quality for tryptophan identification and EOM simulations. For instance, LbAha1 N- and C-terminal domains models presented 100% and more than 97%, respectively, of their amino acids residues on favorable or allowed regions in the Ramachandran plot. These models allowed the localization of the Trp residues in the LbAha1 domains and also to perform rigid body simulations. The modeled domains of LbAha1 are shown in Figure 1A, where the linker between the N- and C-terminal domains is presented by a dotted line. LbAha1 has 9 Trp residues along its structure: 4 in the N-terminal domain and 5 in the C-terminal domain. The relative position of the residues is indicated in the LbAha1 modeled domains (Fig. 1A, dark blue). The first three Trp residues in the N-terminal region of LbAha1, which share high conservation degree with hAha1, are represented by a dashed line since they are in a unstructured region in the yAha1 N-terminal domain template [19].

### Recombinant LbAha1 was Obtained Soluble, with High Purity and Folded

In order to study the biochemical and biophysical properties of LbAha1, the recombinant protein was expressed in the soluble fraction, which allowed its purification (Fig. 1B), as described in the Material and methods section. The secondary and tertiary structures of LbAha1 were inspected by circular dichroism and fluorescence spectroscopy, respectively (Fig. 1C). LbAha1 presented a CD spectrum compatible with those for the proteins containing α-helix (∼17%) and β-sheet (∼31%) conformations, which are similar to the CD spectra of other Aha1 proteins [20], [22]. These amounts are in agreement with those found for the yAha1 N-terminal [19] and hAha1 C-terminal (PDB: 1X53), as well as with the homology models (Fig. 1A).

The local tertiary structure of LbAha1 was investigated by intrinsic fluorescence emission spectroscopy in the absence and presence of large amounts of Gnd-HCl (Fig. 1C, *inset*). The λ_max_ of native and unfolded LbAha1 were 338±1 nm and 349±1 nm, respectively. Since the LbAha1 presents 9 Trp residues, each one contributing to the fluorescence spectrum in a particular way, the <λ> was calculated, resulting in values of 345.9±0.1 nm and 355.6±0.1 nm for the native and unfolded protein, respectively. These results suggest that the recombinant LbAha1 has a folded conformation, once the λ_max_ and <λ> for the native protein was at lower wavelengths than for the protein in the presence of denaturant agent.

### LbAha1 is Shared into Two Domains with Dissimilar Chemical Stabilities

**Figure 2:**
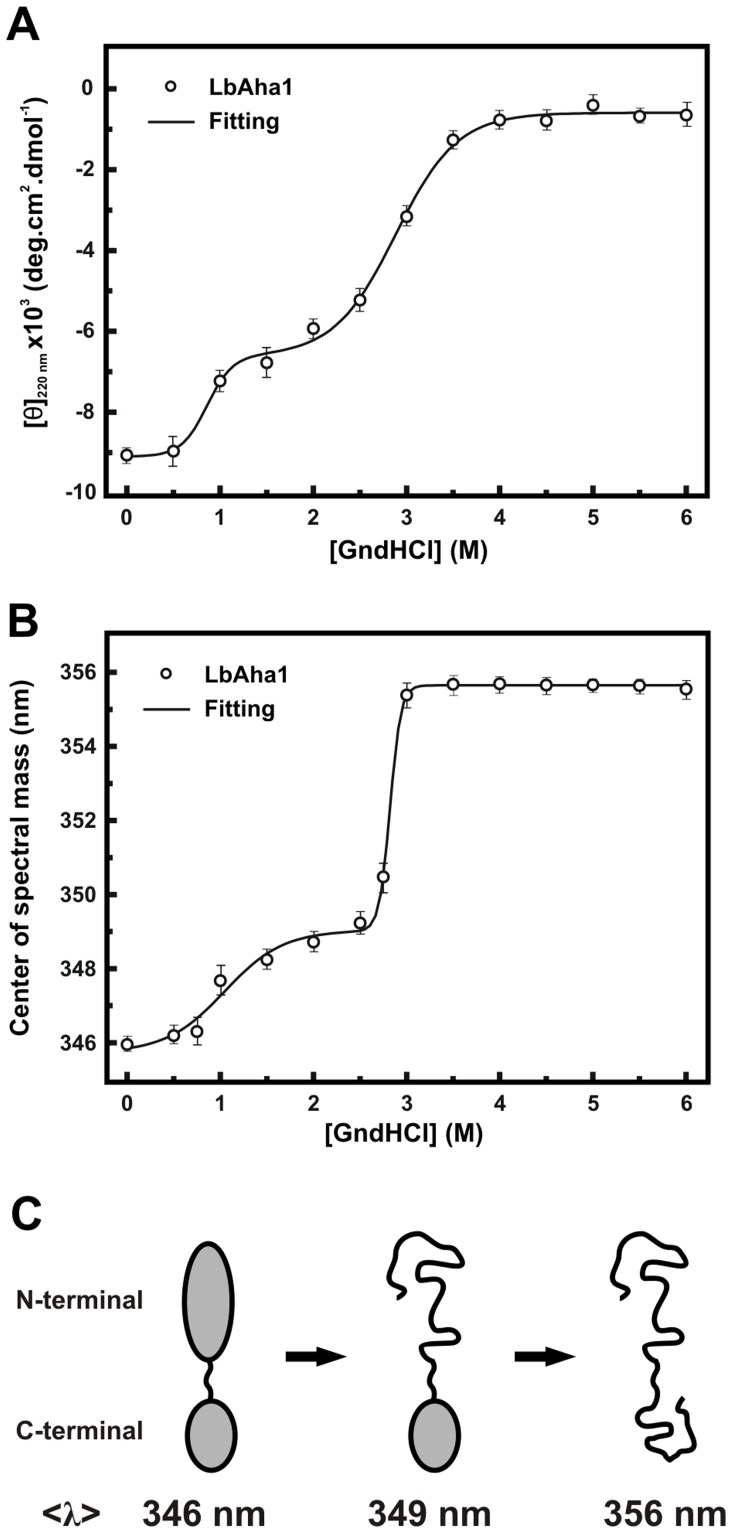
Chemical stability studies of the LbAha1. **A)** The chemical-induced unfolding experiments for LbAha1 were carried out using GndHCl as the denaturing agent and changes in the secondary structure of the protein were monitored by the CD_220nm_ signal, normalized to [θ]. Two defined transitions were observed with C_m_-values centered at 1.0±0.1 and 2.8±0.1 M of GndHCl. **B)** Changes in the tertiary structure of the LbAha1 during its chemical-induced unfolding by GndHCl were monitored by fluorescence. The samples were excited at 280 nm, the fluorescence emission spectra were normalized to <λ>. The C_m_-values determined for each transition were 1.0±0.1 and 2.8±0.1 M of GndHCl. Altogether, these results showed that LbAha1 has two relatively independent domains with different chemical stabilities. **C)** Chemical-unfolding model pathway proposed for LbAha1 based on its structural organization and chemical stabilities (see text for details).

The structural organization of LbAha1 was investigated by means of chemical-induced unfolding experiments using GndHCl as chemical denaturant, and followed by CD at 220 nm (Fig. 2A) and fluorescence spectroscopy (Fig. 2B). With the CD probing, LbAha1 presented two apparent transitions with C_ms_ at 0.9±1 and at 2.9±1 M of GndHCl. Similar results were observed using <λ>-signal as probe with C_ms_ at 1.0±1 and 2.8±1 M of GndHCl. A clear difference holds in the second unfolding transition of the GndHCl-induced unfolding followed by fluorescence, which was more cooperative when compared to the CD_220nm_ curve.

Comparing the denaturant profiles, it was observed that the loss of the secondary structure of LbAha1 occurred almost concomitantly with the changes in the tertiary structure. Moreover, analyzing the amino acid sequences of LbAha1, yAha1 and hAha1 (Fig. S1– Supporting Information), as well as the modeled domains of LbAha1 (Fig. 1A), it was possible to elaborate a hypothesis about the chemical LbAha1 unfolding pathway (Fig. 2C). The LbAha1 N-terminal domain, which has 3 exposed and one partially exposed Trp residues (see Fig. 1A), lost its tridimensional structure first, causing a subtle red-shift in the <λ>-signal (from 346 nm to 349 nm). The 5 tryptophan residues along the LbAha1 C-terminal domain seem to be buried or little exposed to the solvent (see Fig. 1A), resulting in a higher red-shift in the <λ>-signal (from 349 nm to 356 nm) when this domain unfolds. These data corroborate with the dissimilar thermal stabilities of yAha1 isolated domains, suggesting that the yAha1 N-terminal domain is less stable than the C-terminal domain [22].

### LbAha1 Behaves As an Elongated Monomer in Solution

**Figure 3:**
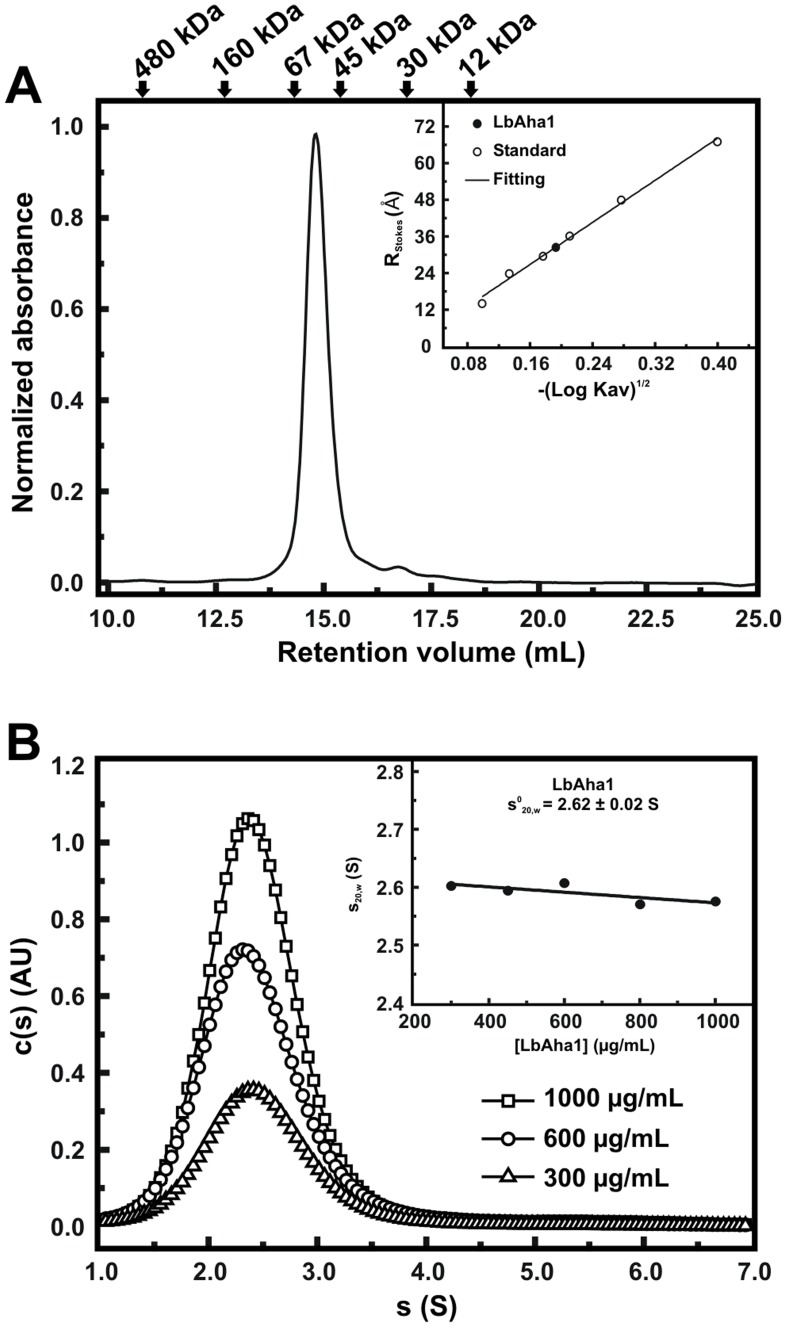
Characterization of the hydrodynamic properties of the LbAha1. **A)** Analytical SEC experiments showed that LbAha1 eluted as one peak between 45 kDa and 67 kDa. The MM of the standard proteins is displayed by arrows. *Inset:* estimation of the LbAha1 Rs, which was 32±2 Å. **B)** Sedimentation velocity experiments showing the c(S) distribution of LbAha1, which behaved as a monomeric species with *s^0^_20,w_* of 2.62±0.02 S, MM of 42±2 kDa and *ƒ/ƒ*_0_ of 1.65±0.04 also at higher concentrations. *Inset*: determination of the *s^0^_20,w_* of the LbAha1 by linear regression analysis. In summary, the LbAha1 protein behaved as an elongated monomer in solution.

To study the hydrodynamic parameters of LbAha1 and therefore to investigate its oligomeric state, shape, among other properties in solution, aSEC and AUC experiments were performed. In the aSEC, LbAha1 eluted as a single peak between globular proteins with MM of 45 kDa and 67 kDa (Fig. 3A). Since the LbAha1 as a monomer presents a theoretical MM of 38 kDa, the elution profile of the protein as a dimer does not explain the observed result. The R_s_ of LbAha1 was then estimated, resulting in a value of 32±2 Å (Fig. 3A, *inset*), which also allowed estimating the *ƒ/ƒ*_0_ of 1.5±0.1 (from the ratio of the R_S_ and R_0_ of a globular protein of 38 kDa). This result suggested that LbAha1 behaves as an elongated monomer in solution. Sedimentation velocity AUC experiments (Fig. 3B) revealed that LbAha1 behaves as a monomeric species with *s^0^_20,w_* of 2.62±0.02 S and MM of 42±2 kDa. Furthermore, the *ƒ/ƒ*_0_ for LbAha1 was 1.65±0.04, a value that also indicated the elongated shape of the protein in solution (Table 1). Taken together, the hydrodynamic properties of LbAha1 are compatible with previous structural studies regarding yAha1 and hAha1, which suggested that they are also elongated monomers in solution [19], [20], [22].

**Table 1:** Summary of the hydrodynamic and structural data for the LbAha1.

Technique		Hydrodynamic and structural properties						
		R_S_ (Å)	R_g_ (Å)	V (×10^−3^ Å^3^)	*ƒ/ƒ*_0_	MM (kDa)	*s^0^_20,w_* (S)	D_max_ (Å)
Predicted data		<$>\raster(65%)="rg1"<$>22	–	¶61	–	38	<$>\raster(65%)="rg1"<$>4.1	–
Analytical SEC		32±2	–	–	□1.5±0.1	–	–	–
AUC		–	–	–	1.65±0.04	42±2	†2.62±0.02	–
SAXS		–	36±2	§70±4	–	47±5	–	140±10
HydroPro	DAM model	34	38	95	–	–	2.59	147
	Selected EOM conformers‡	36±3	37±8	79±2	–	–	2.5±0.2	120±20

^<$>\raster(65%)="rg1"<$>^R_S_ predicted for a hollow sphere with the same MM of the target protein.

¶ Volume estimation based on V_bar_ and MM relationship, considering a hydration of 0.3 g/g.

□ Calculated from the ratio between the experimental and predicted R_S._

† Value of *s^0^_20,w_* determined by linear regression of the AUC data at various protein concentrations.

§ Estimated from Porod’s law.

‡ The values are an average of the HydroPro analyses of 18 EOM selected conformers (Figure S3– see text for details).

### LbAha1 Interacts with LbHsp90 and Stimulates its ATPase Activity Around 10-fold

**Figure 4:**
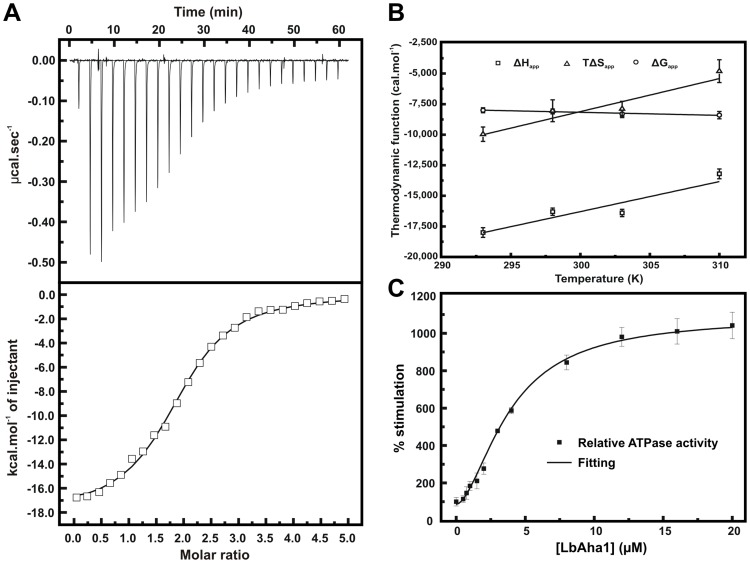
LbAha1 interaction and ATPase activity stimulation of LbHsp90. **A)** The interaction of LbAha1 with the LbHsp90 was investigated by using ITC. The LbAha1 protein was titrated in the LbHsp90, generating a characteristic thermogram of an exothermic reaction. The raw data (upper panel) was treated and the points were adjusted by a One Set of Sites curve fitting (lower panel). These results revealed a LbAha1:LbHsp90 stoichiometry of 2∶1, with a K_D_ of 1.0±0.1 µM and ΔH_app_ of −18,000±400 cal.mol^−1^. **B)** The calorimetric values of ΔH_app_, ΔG_app_ and TΔH_app_ of the binding interaction between LbAha1 and LbHsp90 as a function of temperature. The binding ΔCp_app_ was determined from the slope of ΔH_app_ in dependence on temperature using Equation 3 (see text for details). The black lines represent the linear fitting. **C)** The analysis of the activator effect of LbAha1 on the ATPase activity of LbHsp90 was verified by enzyme kinetics. LbHsp90 (1 µM of dimer) was incubated with LbAha1 at various concentrations (0–16 µM) in 40 mM HEPES buffer (pH 7.5), containing 5 mM KCl and the P_i_ released from ATP hydrolysis was measured spectrophotometrically [16]. The data obtained, treated as relative ATPase activity, presented a sigmoidal behavior and was adjusted using the Hill equation. The results revealed that LbAha1 increased around 10-fold the LbHsp90 ATPase activity in a mechanism of positive cooperativity, with a Hill coefficient of 1.7±0.2.

LbAha1 interaction with LbHsp90 was monitored by means of ITC (Fig. 4A). The dissociation constant obtained was of 1.0±0.1 µM, the same range reported for orthologous Aha1 of human and yeast [19], [22], [41]. The thermodynamic data show an LbAha1:LbHsp90 stoichiometry of 2∶1 (2 molecules of LbAha1 per 1 LbHsp90 dimer), which is in agreement with the model where each Aha1 molecule associates the opposite interfaces in the Hsp90 molecule, at the middle domains. At 20°C, the ΔH_app_ was −18,000±400 cal.mol^−1^ and the calculated ΔS_app_ was −34±2 cal.mol^−1^.K^−1^, suggesting that the LbHsp90-LbAha1 interaction is enthalpically driven and entropy-opposing. These data suggested that the complex formation led to an entropic cost probably due to the freedom degree restriction of LbHsp90 in few conformation states, from several initial conformation states [2].

The interaction of LbAha1 and LbHsp90 was also tested by ITC in the temperature range of 293–310 K, and the thermodynamic data observed are depicted in the Figure 4B. The binding ΔG_app_-values remained constant at the temperatures tested (Fig. 4B) and, based on the Equation 2, the affinity between LbAha1 and LbHsp90 decreased as a function of the temperature. The measured binding ΔH_app_ was largely negative at all temperatures, which drive the interaction, but showed a slightly positive slope as a function of the temperature. Using the Equation 3, which takes into account the dependence on the binding ΔH_app_ and temperature, the binding ΔCp_app_ of +260±80 cal.mol^−1^.K^−1^ was calculated (Fig. 4B). Moreover, the thermodynamic term TΔS_app_, in spite of the negative values at all temperatures tested indicating the entropy-opposite contribution (as asserted above), also presented a slightly positive slope as a function of the temperature (Fig. 4B) in a clear entropy-enthalpy compensation to maintain the binding ΔG_app_ independent on the temperature. The binding ΔCp_app_ is an important thermodynamic parameter that can supply information about the interaction mechanism and positive binding ΔCp_app_ have been commonly attributed to hydration events, hydrogen bounds and/or long-range electrostatic interactions arisen from binding of the interactors [42], [43]. Interestingly, the co-crystallographic structure of yAha1 N-terminal and yHsp90 MD (PDB: 1USV) exhibits an extensive network contacts involving amino acids that interact by electrostatic interactions and hydrogen bounds, including water-mediated interactions [19]. Therefore, interactions involving charged amino acids and hydrogen bounds may also have considerable contribution to the interaction between LbAha1 and LbHsp90. The sequence analysis of the LbAha1 showed that, in the known mapped regions of yAha1 that interact with yHsp90 MD, the identity and similarity between LbAha1 and yAha1 are 28% and 51%, respectively; values slightly higher than in the rest of the proteins (Fig. S1B – Supporting Information). These characteristics suggest that LbAha1::LbHsp90 interaction may present similar molecular mechanism to the yAha1::yHsp90 system, despite the low conservation degree, in the amino acid sequence, between LbAha1 and yAha1 (Fig. S1B – Supporting Information).

It is known that Aha1 proteins are able to interact with Hsp90 and stimulate its ATPase activity [17], [20]. The maximum stimulation of Hsp90 ATPase activity occurs with the full length Aha1, although some studies reported that a substantial activation of Hsp90 could be achieved with the N-terminal domain alone [17], [22]. The precise role of the C-terminal domain is still controversial, but it is suggested that additional contacts between the Aha1 C-terminal and Hsp90 N-domain participate in the Hsp90 activation in a cooperative way with the Aha1 N-terminal domain [20], [22].

Enzyme kinetic experiments were performed to check the ability of the LbAha1 to stimulate the LbHsp90 ATPase activity. We have shown that LbHsp90 has a weak ATPase activity (0.320 min^−1^), which is in the same range of both yHsp90 and hHsp90 [16]. The results indicated that LbAha1 stimulated LbHsp90 ATPase activity around 10-fold (Fig. 4C). Furthermore, the enzyme kinetics curve presented a sigmoidal format, suggesting a cooperative behavior among LbAha1-LbHsp90 interactions. The fitting of the curve data using the Hill equation resulted in a Hill coefficient of 1.7±0.2, which represents a mechanism of positive cooperativity for LbAha1 stimulating the LbHsp90 ATPase activity. This behavior was observed in previous reports for hHsp90, although it has never been undertaken [20], [44]. A dissociation constant of 1–2 µM has been described for the Aha1-Hsp90 interaction [17], [20], [22] (see above) and, in saturated conditions, it was reported that yAha1 can bind to yHsp90 in a 2∶1 stoichiometry [22], as also shown for LbAha1 by ITC experiments. Within this context, the functional cooperativity effect seen on the LbHsp90 ATPase activity experiments could be due to the interactions among two units of LbAha1 with one unit of LbHsp90 (2 monomers of LbAha1 per 1 dimer of LbHsp90), considering the high molar ratios achieved in these kind of experiments.

### Low Resolution Structural Models Based on SAXS Data Show the LbAha1 Structural Organization as a Highly Elongated Monomer

**Figure 5:**
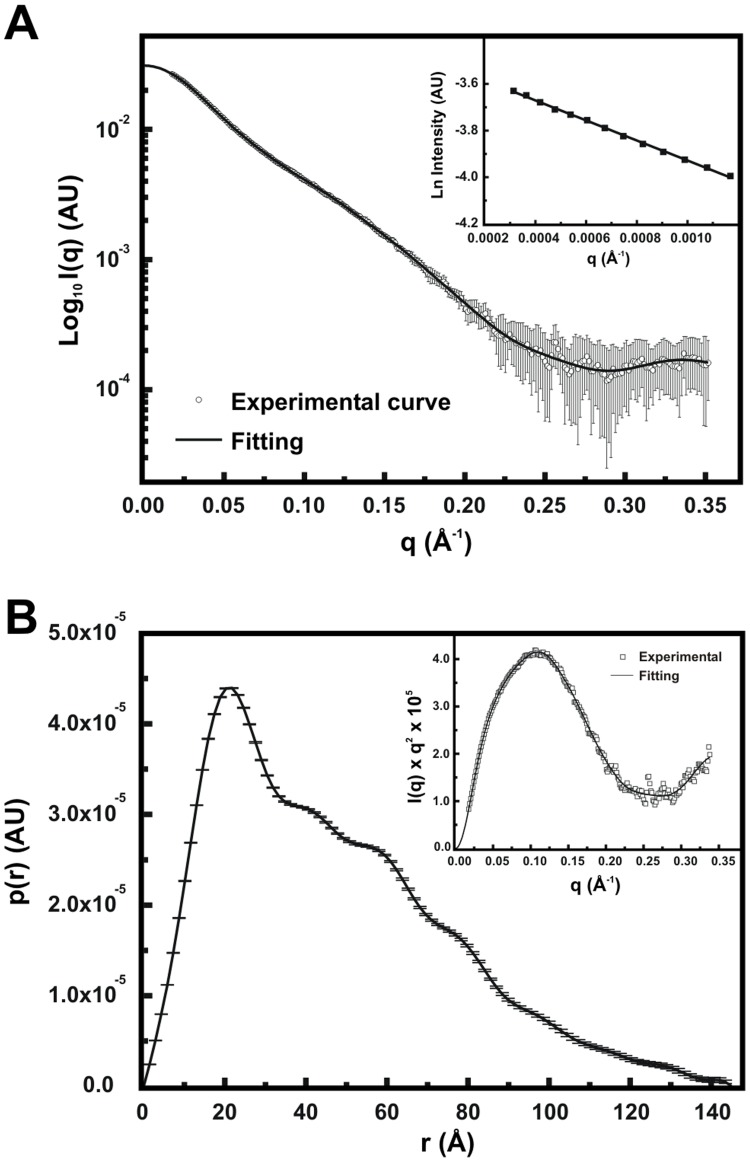
LbAha1 SAXS curves and data analysis. **A)** The final scattering curve (open circles) of the LbAha1 was the average of the SAXS curves collected at various protein concentrations. The solid line represents the SAXS curve fitting until q = 0 Å^−1^ performed by the GNOM program during the distance distribution function generation. *Inset*: Guinier analysis of the LbAha1 SAXS curve showing its linearity. **B)** The particle distance distribution function of the LbAha1 was constructed by the GNOM program using the SAXS curve shown in **A**). LbAha1 showed a D_max_ of 140±10 Å and the p(r) revealed a prolate shape. *Inset:* Kratky plot showing that LbAha1 is a compact protein.

The available structural information on Aha1 proteins comes from crystallographic and NMR studies with the yAha1 N-terminal domain [19] and the hAha1 C-terminal domain (PDB ID: 1X53– unpublished data). However, these studies investigated the structure of the individual domains of Aha1, yet none of them revealed how the entire protein behaves in solution. To address this question, SAXS experiments were performed with LbAha1 (Fig. 5) and low resolution structural models were reconstructed. The Guinier region of the LbAha1 scattering curve was analyzed, and displayed no features that characterize protein aggregation (Fig. 5A, *inset*), yielding R_g_ of 36±2 Å. Moreover, using the *I*(0), the value of LbAha1 MM was estimated in 47±5 kDa, in agreement with a monomeric LbAha1. Figure 5B depicts the LbAha1 p(r) distribution function pointing a D_max_ of 140±10 Å and a curve shape similar to those of prolate-shaped particles [45]. In addition, the Kratky plot indicated that LbAha1 do not have random or unfolded structure. Since LbAha1 consist of two domains with different stabilities, they should present a folded and compact structure probably linked by a flexible linker (Fig. 5B, *inset*). These data are in good agreement with LbAha1 as an elongated monomer, as observed in the hydrodynamic results presented above.

**Figure 6:**
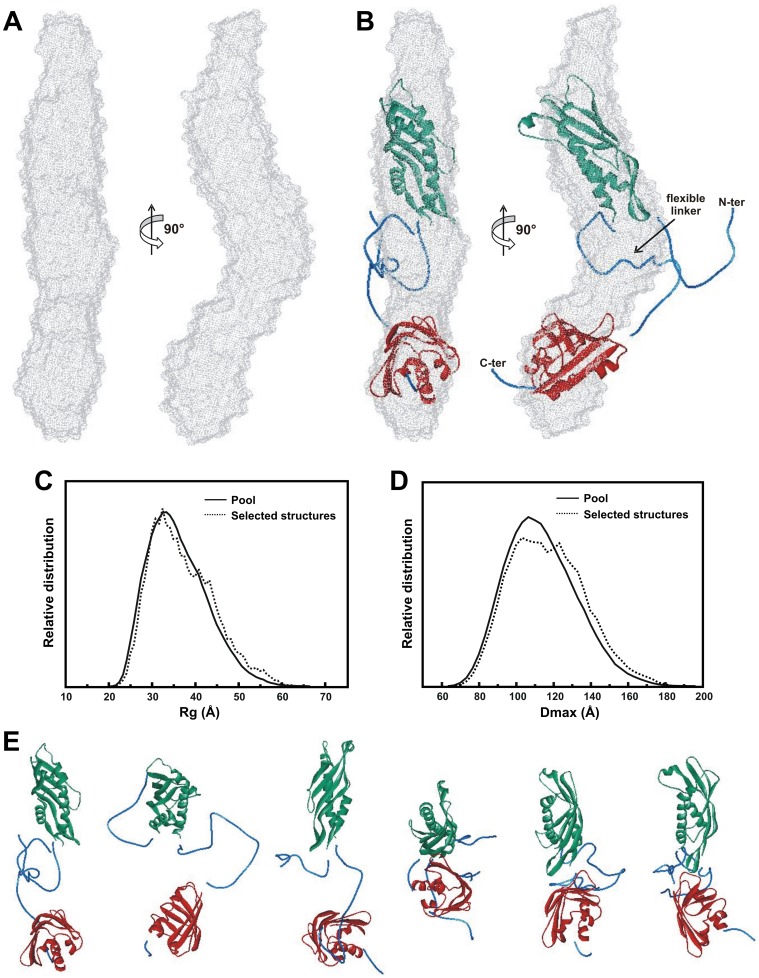
Low resolution structure and rigid body modeling of the LbAha1. **A)** DAM model and **B)** LbAha1 EOM conformer (superposed to the DAM model) in different views displaying its domain arrangement and the relative independence between them. This EOM conformer, which is one of the 18 selected conformers (Figure 6E and Figure S3– Supporting Information) of the best ensemble simulated for LbAha1, was selected based on the best superposition with the shape of the DAM model. **C)** R_g_ and **D)** D_max_ relative distributions of the LbAha1 conformers calculated by the EOM approach considering the experimental SAXS pattern. The solid lines represent the distribution within the pool of 10,000 conformers and the dotted lines, the R_g_- and D_max_-values observed for the best ensemble conformers. **E)** Representative LbAha1 conformers present in the best ensemble obtained by the EOM. The first conformer was used for superposing to the DAM model in the panel B. The N-terminal and C-terminal domains are shown in green and red, respectively; the reconstructed missing regions are displayed in blue.

Based on the SAXS data, an LbAha1 *ab initio* model was reconstructed using the DAMMIN program (see Material and methods section for details). A total of 12 independent reconstructions were ran and the DAMAVER suite was used to average the models, considering only *ab initio* models with low NSD value (normalized spatial discrepancy), as follows: NSD<Mean +2× Variation (Mean = 0.552; Variation = 0.029). The merged DAM model is shown in Figure 6, which emphasizes the LbAha1 elongated shape. This model was validated by comparing its predicted hydrodynamic properties, evaluated by the HydroPro program, with the experimental data. The values found were in excellent agreement with the experimental hydrodynamic data obtained for LbAha1 protein (Table 1).

Rigid body fitting based on SAXS data is an attractive strategy for interpreting the inner organization of multi-domain proteins when high resolution structural models of the individual domains are available [46], [47]. Due to the presence of a linker between the LbAha1 domains, the domain simulation was performed using the EOM approach [39], [47] and the homology models generated for LbAha1 domains. The EOM strategy takes into account the flexibility and allows the coexistence of different conformers that contribute to the final scattering pattern. It is important to mention that SAXS is a low resolution technique and this simulation considers the scattering amplitude of each atom to calculate the SAXS pattern to position both domains spatially. The EOM routine generated 10,000 random conformers covering the LbAha1 configurational space and selected 18 conformers (Fig. S3– Supporting Information), which represent an ensemble of the LbAha1 conformers in solution, based on goodness-of-fit of the ensemble conformers to the experimental scattering pattern (Fig. S2– Supporting Information). All selected LbAha1 conformers were also submitted to the HydroPro analysis, as well as the DAM model, and presented hydrodynamic and structural properties that matched with the experimental ones (Table 1), which also validate the ensemble conformers. One of them is presented superimposed into the envelope of the DAM model and showed a good match (Figure 6B). As asserted above, the EOM routine takes into account the flexibility of the protein since SAXS data is a temporal scattering average of all conformations of all scattering particles. Therefore, structural properties like R_g_ and D_max_ of the EOM conformers should be similar to the experimental values. The Figure 6C-D shows a broad R_g_ and D_max_ distribution values and compared to the pool of 10,000 EOM conformers, the selected ensemble presents a similar Gaussian distribution. These data indicate that the LbAha1 linker is almost completely flexible and does not interfere with the relative orientation of both N- and C-terminal domains. Once the selected ensemble presents a scattering pattern that showed a good match to the experimental scattering data, the conformers have consistent size/shape to the population of LbAha1 molecules in the solution. Interestingly, the LbAha1 hydrated volume was estimated from SAXS data and it was similar to that calculated for a hydrated particle of 38 kDa (Table 1). However, the hydrated volumes estimated for EOM molecules as well as for DAM model were slightly higher and could be related to the protein flexibility. These data indicate that LbAha1 domains may present a distention-contraction conformation changes and are interconnected by a flexible linker.

Interestingly, SAXS data analysis of LbAha1 confirmed its elongated shape in solution, revealing its domains arrangement, also suggesting a relative independence of both C- and N-terminal domains. Furthermore, the LbAha1 low resolution models presented a coherent shape with the mechanism of interaction proposed between Aha1 and Hsp90 proteins [10]. In this model, the Aha1 N-terminal domain interacts with the Hsp90 MD and additional contacts are made between the Aha1 C-terminal domain and the dimerized ND of Hsp90, inducing conformational changes that facilitate the ATP hydrolysis in the Hsp90 [10], [20], [22]. Furthermore, the D_max_ observed for the C-domain deletion mutant of LbHsp90 was 120±20 Å (unpublished data), which fits well with the D_max_ of 140±10 Å observed for LbAha1, allowing those proteins to interact.

### Aha1 is a Cognate Protein in Three Leishmania Species

**Figure 7:**
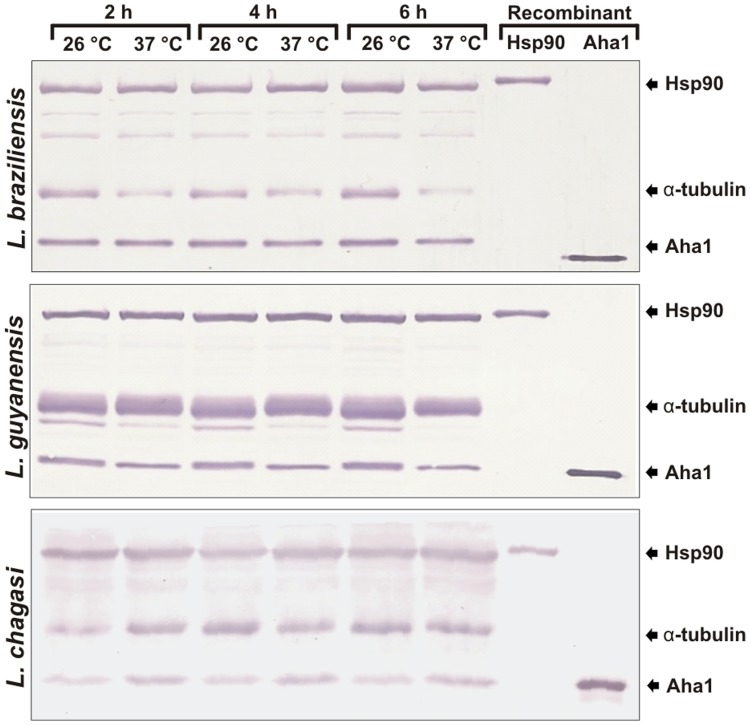
Western blotting analysis of Aha1 and Hsp90 molecular chaperones. The expression levels of LbAha1 and LbHsp90 proteins were analyzed in three *Leishmania* species: *L. braziliensis*, *L. guyanensis* and *L. chagasi* cultured at 26°C or 37°C during 2, 4 or 6 h. Rabbit polyclonal antibodies anti-Aha1 and anti-Hsp90 recognized the native and recombinant proteins. Monoclonal anti-α-tubulin antibody was used as loading control. The arrows indicate the protein bands.

The presence and importance of Hsp90 in trypanosomatids has been shown [3]–[6]. However little is known about the Hsp90 co-chaperones, including their *in vivo* presence and roles. To identify and investigate the expression profile of the Aha1 protein in function of time and temperature, western blot analysis was carried out using three *Leishmania* species: *L. braziliensis*, *L. guyanensis* and *L. chagasi* incubated at 26°C or 37°C. Western blotting results showed that the anti-LbAha1 and anti-LbHsp90 antibodies recognized native proteins with expected size of 38 kDa and 90 kDa, respectively (Fig. 7). As controls, *L. braziliensis* recombinant proteins were used as standards, both Aha1 and Hsp90 proteins were also identified. Surprisingly, the Aha1 proteins were found being expressed at normal parasite growth conditions (26°C) and at heat-shock conditions (37°C). These results clearly show that Aha1 is a cognate and constitutively expressed protein, as well as the Hsp90 molecular chaperone.

### Conclusions

We produced the recombinant LbAha1 protein and performed several experiments in order to characterize its structure-function relationship. To the best of our knowledge this is the first work that has characterized the Aha1 protein of a trypanosomatid organism. We identified Aha1 as well as Hsp90 proteins in three Leishmania species, including *L. braziliensis*. The protein expression levels were similar in parasites incubated at 26 ^o^C and 37 ^o^C, suggesting that Aha1 is a cognate protein in protozoa. This is interesting data, since there is increasing interest regarding the Hsp90 molecular chaperone family of protozoa as a target for inhibition and treatment of protozoa diseases [4], [48]. According to the World Health Organization, Leishmaniasis is a neglected disease. It is estimated that worldwide 12 million people are infected and 350 million are at risk of infection [23], [24], representing a challenge to health care, including the need for new medicines [25].

It has been shown that orthologous Aha1 are modular proteins where the N-terminal domain perform the main interaction contact with the Hsp90 MD helping to align the Arg380 with the γ-phosphate of ATP [19]. However, the role of the C-terminal, which influences the Hsp90 ATPase activity, remained in second plan. Two recent works have shown that the Aha1 C-terminal domain directly interacts with the Hsp90 ND [20], [22]. Here, we observed that for LbAha1 these two important structural characteristics are critical for its functional activity: 1) two relatively independent domains connected by a flexible linker, and 2) highly elongated shape. The dimension observed allows LbAha1 to interact with both Hsp90 ND and Hsp90 MD, either symmetrically as well as asymmetrically, while inducing a dual effect. The domain independence pointed out that LbAha1 is a modular protein which allows the Aha1 C-terminal domain to dock on the Hsp90 ND after the first contact has been established between Aha1 N-terminal and Hsp90 MD. These characteristics allow LbAha1 (and orthologous proteins) to synergistically act on stimulating the Hsp90 ATPase activity by influencing both Arg380 (numbering of yHsp90) in the Hsp90 MD and some catalytic residues in the Hsp90 ND.

Functionally, LbAha1 interacted with LbHsp90 dimer in a 2∶1 stoichiometry and with dissociation constant at the micromolar range. The thermodynamic data also indicate that the interaction was guided by enthalpy and with an entropy-opposing interaction probably due to the freedom degree restriction of LbHsp90. Moreover, LbAha1 stimulated LbHsp90 ATPase activity 10-fold in a sigmoidal curve suggesting a positive cooperative mechanism on the LbHsp90 ATPase activity stimulation. Taken all together, LbAha1 shares several structural and functional properties with the human and yeast orthologues, suggesting similar functional mechanism among these proteins despite the low conservation degree in the amino acid sequence.

## Supporting Information

Figure S1 **Sequence alignment analysis of LbAha1.** The LbAha1 was compared with the hAha1 (**A**) and yAha1 (**B**) proteins in order to investigate its evolutionary conservation. LbAha1 shares 30/49% and 23/41% of identity/similarity with hAha1 and yAha1, respectively. These values indicate a low conservation among the amino acid sequences, which could lead to peculiarities in the mechanism of action of the LbAha1 in the LbHsp90 ATPase activity stimulation. The red boxes represent the already indentified amino acids involved in the yAha1 N-terminal domain interaction with the yHsp90 MD (PDB: 1USV). In these regions, LbAha1 presents 28/51% of identity/similarity to the yAha1 protein, slightly higher than in the rest of the protein sequences. The N- and C-terminal domains are underlined, and the linker region is indicated by the box. The conserved amino acid residues (*), the residues with strongly similar properties (:) and the residues with weakly similar properties (.) are showed.
(TIF)

Figure S2 ***Ab initio*** **DAMMIN and EOM simulations from small angle X-ray scattering data.** **A)** The DAMMIN program generated the *ab initio* models of the LbAha1 by adjusting the simulated curve to the experimental SAXS curve in a simulated annealing method. The average of the simulated curves of the *ab initio* models used to reconstruct the low resolution structure of the LbAha1 is showed (solid line), as well as the experimental SAXS curve (open circles). The averaged χ for these adjustments was 1.34±0.08. **B)** The EOM routine was used the RanCh program to create 10,000 random LbAha1 models and the best ensemble based on the experimental SAXS curve of the protein (open circles) was selected by the GAJOE program. The ensemble with the better curve adjustment (solid line) was selected and presented a χ-value of about 1.96.
(TIF)

Figure S3 **The best LbAha1 conformers generated by the EOM routine.** The RanCh program generated 10,000 random models of LbAha1, from which 18 models were selected by the GAJOE program based on the fitting goodness to the experimental SAXS curve (Fig. S2B). All the models above represent some possible LbAha1 conformers that reflect the experimental SAXS data. The N- and C-terminal domains are showed in green and red, respectively. The missing regions reconstructed by the RanCh program are shown in blue.
(TIF)
