# Supplementary figures and images for: Low Resolution Structural Studies Indicate that the Activator of Hsp90 ATPase 1 (Aha1) of Leishmania braziliensis Has an Elongated Shape Which Allows Its Interaction with Both N- and M-Domains of Hsp90
Source: PLoS One. 2013 Jun 24;8(6):e66822. doi: 10.1371/journal.pone.0066822 (PMC3691308; doi:10.1371/journal.pone.0066822)

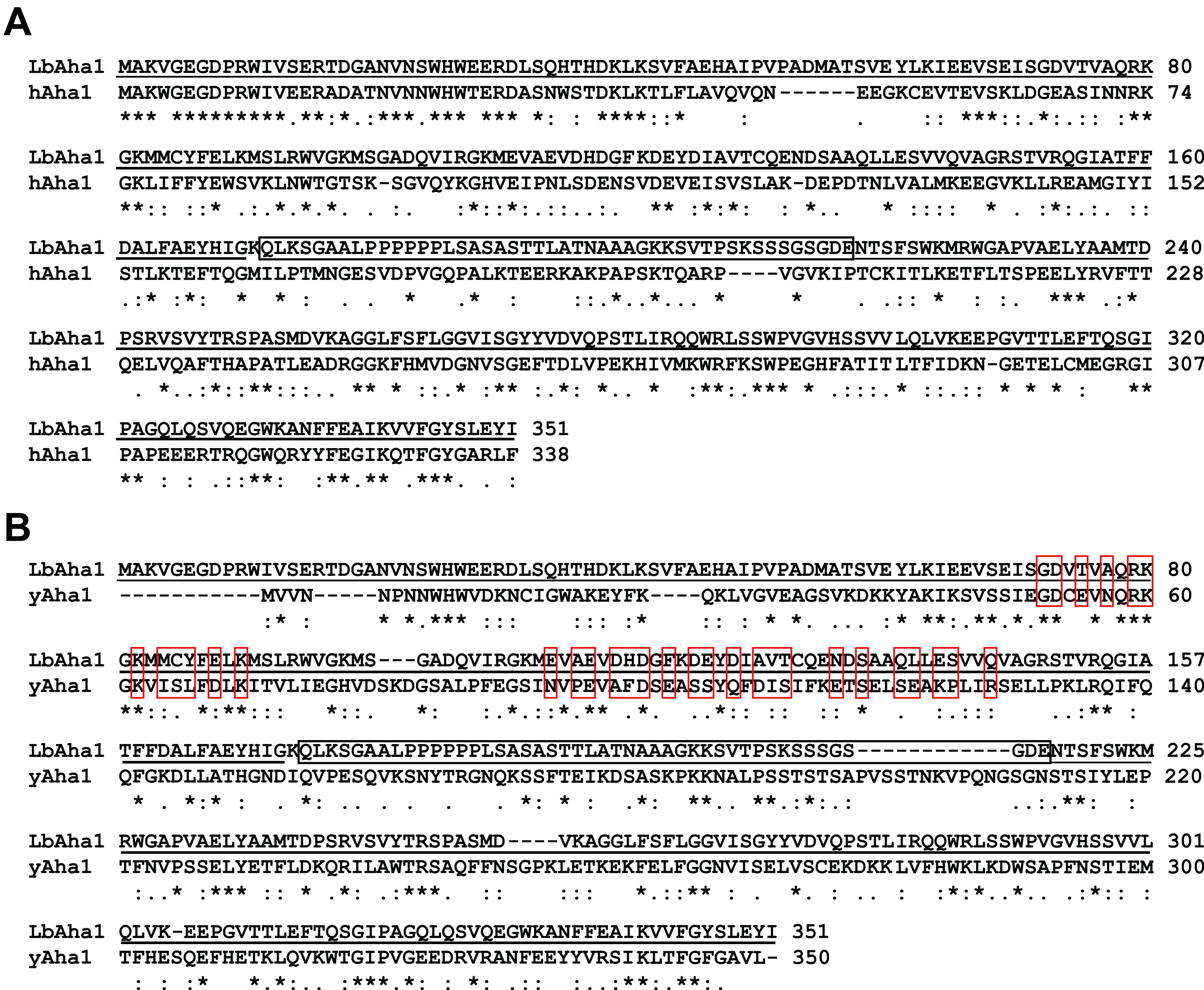

Supplement: Figure S1 — Sequence alignment analysis of LbAha1. The LbAha1 was compared with the hAha1 (A) and yAha1 (B) proteins in order to investigate its evolutionary conservation. LbAha1 shares 30/49% and 23/41% of identity/similarity with hAha1 and yAha1, respectively. These values indicate a low conservation among the amino acid sequences, which could lead to peculiarities in the mechanism of action of the LbAha1 in the LbHsp90 ATPase activity stimulation. The red boxes represent the already indentified amino acids involved in the yAha1 N-terminal domain interaction with the yHsp90 MD (PDB: 1USV). In these regions, LbAha1 presents 28/51% of identity/similarity to the yAha1 protein, slightly higher than in the rest of the protein sequences. The N- and C-terminal domains are underlined, and the linker region is indicated by the box. The conserved amino acid residues (*), the residues with strongly similar properties (:) and the residues with weakly similar properties (.) are showed. (TIF) [file pone.0066822.s001.tif]

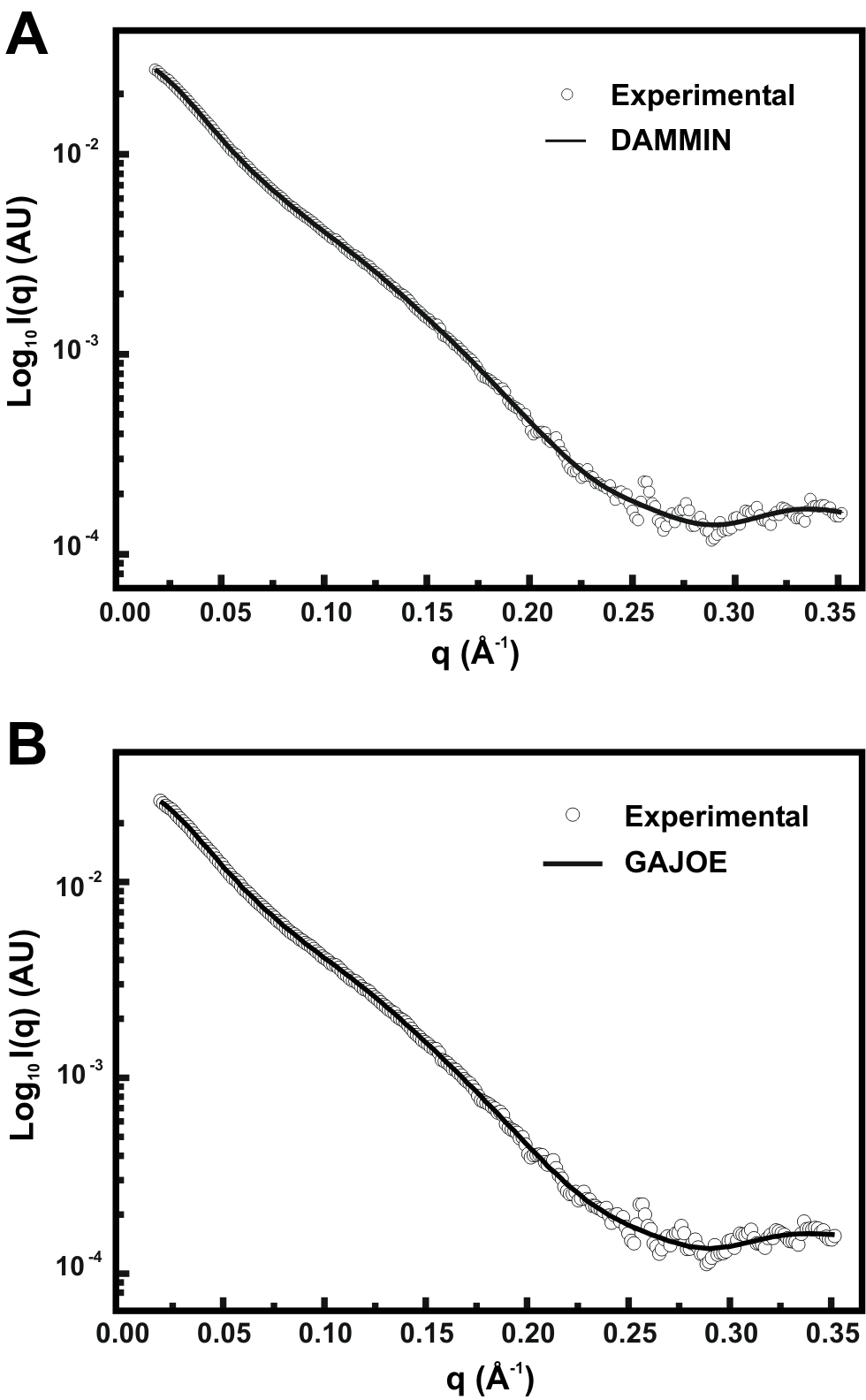

Supplement: Figure S2 — Ab initio DAMMIN and EOM simulations from small angle X-ray scattering data. A) The DAMMIN program generated the ab initio models of the LbAha1 by adjusting the simulated curve to the experimental SAXS curve in a simulated annealing method. The average of the simulated curves of the ab initio models used to reconstruct the low resolution structure of the LbAha1 is showed (solid line), as well as the experimental SAXS curve (open circles). The averaged χ for these adjustments was 1.34±0.08. B) The EOM routine was used the RanCh program to create 10,000 random LbAha1 models and the best ensemble based on the experimental SAXS curve of the protein (open circles) was selected by the GAJOE program. The ensemble with the better curve adjustment (solid line) was selected and presented a χ-value of about 1.96. (TIF) [file pone.0066822.s002.tif]

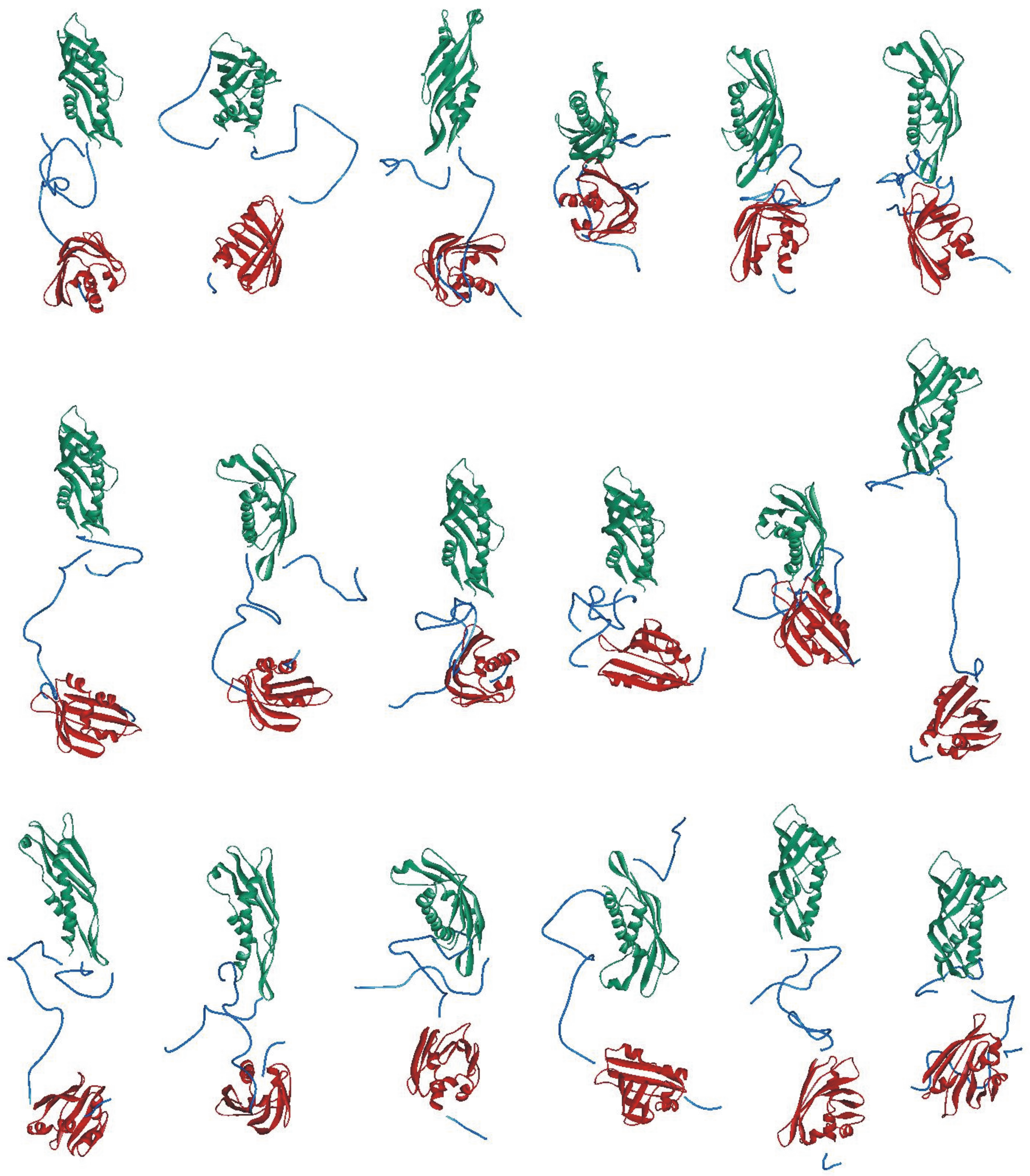

Supplement: Figure S3 — The best LbAha1 conformers generated by the EOM routine. The RanCh program generated 10,000 random models of LbAha1, from which 18 models were selected by the GAJOE program based on the fitting goodness to the experimental SAXS curve (Fig. S2B). All the models above represent some possible LbAha1 conformers that reflect the experimental SAXS data. The N- and C-terminal domains are showed in green and red, respectively. The missing regions reconstructed by the RanCh program are shown in blue. (TIF) [file pone.0066822.s003.tif]
